# The immunity priming effect of the *Arabidopsis* phyllosphere resident yeast *Protomyces arabidopsidicola* strain C29

**DOI:** 10.3389/fmicb.2022.956018

**Published:** 2022-09-02

**Authors:** Kai Wang, Agate Auzane, Kirk Overmyer

**Affiliations:** Organismal and Evolutionary Biology Research Program, Faculty of Biological and Environmental Sciences, and Viikki Plant Science Centre, University of Helsinki, Helsinki, Finland

**Keywords:** beneficial microbes, *Botrytis*, defenses against pathogens, MAPK, plant–fungal interactions, Protomycetaceae, Taphrinomycetes, yeast-like fungi

## Abstract

The phyllosphere is a complex habitat for diverse microbial communities. Under natural conditions, multiple interactions occur between host plants and phyllosphere resident microbes, such as bacteria, oomycetes, and fungi. Our understanding of plant associated yeasts and yeast-like fungi lags behind other classes of plant-associated microbes, largely due to a lack of yeasts associated with the model plant *Arabidopsis,* which could be used in experimental model systems. The yeast-like fungal species *Protomyces arabidopsidicola* was previously isolated from the phyllosphere of healthy wild-growing *Arabidopsis*, identified, and characterized. Here we explore the interaction of *P. arabidopsidicola* with *Arabidopsis* and found *P. arabidopsidicola* strain C29 was not pathogenic on *Arabidopsis*, but was able to survive in its phyllosphere environment both in controlled environment chambers in the lab and under natural field conditions. Most importantly, *P. arabidopsidicola* exhibited an immune priming effect on *Arabidopsis*, which showed enhanced disease resistance when subsequently infected with the fungal pathogen *Botrytis cinerea*. Activation of the mitogen-activated protein kinases (MAPK), camalexin, salicylic acid, and jasmonic acid signaling pathways, but not the auxin-signaling pathway, was associated with this priming effect, as evidenced by MAPK3/MAPK6 activation and defense marker expression. These findings demonstrate *Arabidopsis* immune defense priming by the naturally occurring phyllosphere resident yeast species, *P. arabidopsidicola,* and contribute to establishing a new interaction system for probing the genetics of *Arabidopsis* immunity induced by resident yeast-like fungi.

## Introduction

The phyllosphere is the above ground external surface environment of plants and a complex habitat for microbes. The relationships of phyllosphere microbial residents with host plants can be pathogenic, mutualistic, or frequently commensal. This growth space represents a significant reservoir for latent pathogens, the ecological role of which remains poorly defined (Jarvis, 1994; Saikkonen et al., 1998; Slippers and Wingfield, 2007). Plant beneficial effects induced by plant-associated microbes, such as protection against pathogens and growth promotion, have gained attention recently. Priming is the process of initiating enhanced immune defense responses upon encountering pathogenic microbes, non-pathogenic microbes, or a variety of compounds, both natural and synthetic (Balmer et al., 2015; Conrath et al., 2015). Systemic-acquired resistance activated by necrotizing pathogens, as well as induced systemic resistance triggered by yeast-like fungi, filamentous fungi, and rhizobacteria in soil, are believed to be associated with priming of defense responses (Conrath et al., 2006, 2015; Hossain et al., 2007; Buxdorf et al., 2013a; Balmer et al., 2015; Narusaka et al., 2015). Signaling pathways involving hormones, mRNA, protein kinases, transcriptional factors, and epigenetic regulation all have demonstrated roles in plant immune defense (Beckers et al., 2009; Pieterse et al., 2014).

Yeasts and yeast-like fungi modulate plant growth and health (Nassar et al., 2005; Nafady et al., 2019; Mukherjee et al., 2020). Plant growth-promoting traits and induction of defense response by yeasts occur in both cultivated plants and postharvest fruits (Bergauer et al., 2005; Liu et al., 2013; Nutaratat et al., 2014). Several yeasts with so-called pathogen antagonistic activity have the ability to suppress plant diseases caused by various pathogenic fungi (Liu et al., 2013; Mukherjee et al., 2020; Eitzen et al., 2021). Yeasts, such as *Candida*, *Pichia*, and *Cryptococcus* species, inhibit fungal invasion in postharvest biocontrol systems through induction of host defense (Droby et al., 2002; El Ghaouth et al., 2003; Chan et al., 2007; Zheng and Chen, 2009; Macarisin et al., 2010; Xu et al., 2013). Thus, some yeasts are used as bio-agents for promotion of plant growth and immunity in sustainable agriculture (Liu et al., 2013; Mukherjee et al., 2020).

Several plants exhibit multiple responses to various yeast elicitor preparations; such as autoclaved baker’s yeast (*Saccharomyces cerevisiae*), preparations from *S. cerevisiae* cell walls, or commercial yeast extract. These responses include, increased immunity against pathogens, induction of reactive oxygen species accumulation, and activation of known immune signaling pathways, in the model plant *Arabidopsis thaliana* (referred to hereafter as *Arabidopsis*; Raacke et al., 2006; Khokon et al., 2010; Minami et al., 2011; Ye et al., 2013; Narusaka et al., 2015; Yaguchi et al., 2017) and other plants (Sun et al., 2018a, 2018b). Raacke et al. (2006) used reverse genetics with *Arabidopsis* to demonstrate that yeast treatments induced salicylic acid (SA) dependent immunity against *Pseudomonas syringae*; however, induced resistance against the fungal pathogen *Botrytis cinerea* (hereafter referred to as *Botrytis*) was independent of the SA, jasmonate, and camalexin pathways. Although immune priming occurs in *Arabidopsis* treated with various yeast species isolated from other plants (Buxdorf et al., 2013a,b; Ferreira-Saab et al., 2018), a specific interaction system with a naturally occurring phyllosphere resident yeast and the model plant *Arabidopsis* has not been established.

*Protomyces* species are yeast-like members of the subphylum Taphrinomycotina (Ascomycotina; Reddy and Kramer, 1975; Kurtzman, 2011). *Protomyces* species are all dimorphic; they exist in a non-infectious haploid resident yeast phase and infect their hosts as dikaryotic hyphal pathogenic phase after conjugating with another cell of a compatible mating (MAT) type. Nearly all members of this genus are plant pathogens, mostly of wild plant species, but some crops are affected. For instance, *P. macrosporus,* which causes coriander stem-gall disease in many regions, threatens coriander seed production (Pavgi and Mukhopadhyay, 1972; Malhotra et al., 2016; Khan and Parveen, 2018). Species in the genus *Protomyces* cause symptoms such as tumors or galls on flowers, leaf veins, petioles, and stems (Reddy and Kramer, 1975; Kurtzman, 2011; Streletskii et al., 2019). Until recently, the definition of the genus *Protomyces* encompassed only species pathogenic of host plants within the families Asteraceae and Apiaceae (Reddy and Kramer, 1975; Kurtzman, 2011). However, a phyllosphere resident *Protomyces* species was isolated from healthy wild growing *Arabidopsis*, characterized, and named *Protomyces arabidopsidicola* (type strain C29; Wang et al., 2016, 2021). Genomic, morphological, and physiological features of *P. arabidopsidicola* differentiate it from other described *Protomyces* species. Its occurrence on *Arabidopsis*, a member of the Brassicaceae, suggests *Protomyces* host-range may not be restricted only to the Asteraceae and Apiaceae families (Wang et al., 2021). However, the relationship of *P. arabidopsidicola* with *Arabidopsis* remains undetermined.

Our understanding of plant-associated yeasts lags behind that of other classes of microbes. This is especially true for yeasts associated with *Arabidopsis*, a model plant widely used when studying the molecular basis of plant-microbe interactions (Boutrot and Zipfel, 2017). Plants have a two tiered immune system (Jones and Dangl, 2006), with pattern recognition receptors (PRRs) that perceive pathogen associated molecular patterns (PAMPs), which are also present in non-pathogenic microbes and thus are also often referred to as microbe associated molecular patterns (MAMPs). PAMP perception leads to activation of basal immunity, termed PAMP-triggered immunity (PTI), which limits infections by pathogens not specifically adapted to that plant. Adapted pathogens overcome PTI by deploying small-secreted proteins and toxins, termed effectors. In turn, plants counter effectors with nucleotide-binding, leucine-rich repeat immune receptors, which perceive effector action to activate immune responses. The dynamics of plant immune function in a molecular evolutionary arms race between host and pathogen is summarized in the zigzag model (Jones and Dangl, 2006; Pritchard and Birch, 2014).

Plants and metazoans share a structurally and functionally conserved innate immune system (Nurnberger and Brunner, 2002; Nurnberger et al., 2004). Although the receptors and mechanisms of perception differ, plants and metazoans have independently evolved the ability to perceive many of the same PAMPs using receptors with similar conserved-domain architectures (Nurnberger and Brunner, 2002; Nurnberger et al., 2004). The human immune system includes a complement of PRRs that specifically recognize PAMPs from yeast cell walls (Jouault et al., 2009; Saijo et al., 2010; Patin et al., 2019). Plants have the ability to perceive some molecules present in yeast cell walls, such as chitin, mannopeptides, and β-glucans, which function as PAMPs (Hahn and Albersheim, 1978; Basse et al., 1992; Felix et al., 1993; De Jonge et al., 2010; Mélida et al., 2018; Christita et al., 2021). However, most of the plant receptors responsible for detecting these PAMPs remain unidentified or are not well characterized. Only chitin has a well-described set of PRRs in plants (Faulkner et al., 2013; Xue et al., 2019). *Arabidopsis* perceives chitin with receptor complexes containing members of the lysin motif (LysM) receptor kinases; LYK1 (also known as CERK1), LYK2, LYK4, and LYK5 (Miya et al., 2007; Wan et al., 2008; Faulkner et al., 2013; Cao et al., 2014; Xue et al., 2019). Additionally, CERK1 is required for perception of linear β-1,3-glucan (Mélida et al., 2018). The LysM domain is responsible for chitin binding and is found in both plant chitin receptors and fungal effector proteins that bind and sequester chitin to suppress immunity (Doehlemann and Hemetsberger, 2013).

Microbe recognition triggers mitogen-activated protein kinase (MAPK) activation. This is a key early event in PAMP-induced immune signaling. MAPK3 and MAPK6 are differentially required for immunity against the broad host-range fungal pathogen *Botrytis* induced by oligogalacturonides or flagellin (Galletti et al., 2011). In addition, perception of the fungal PAMP chitin results rapid activation of a MAPK cascade in *Arabidopsis* and rice (Miya et al., 2007; Wan et al., 2008; Yamada et al., 2017). MAPK activation kinetics can vary greatly, lasting for minutes to hours, depending on the MAPK involved and the activating stimulus (Bigeard and Hirt, 2018). The plant immunity network also involves complex and interconnected hormone signaling pathways, including SA, jasmonic acid (JA), ethylene, and others. Additionally, many microbes, including *Protomyces* are known to produce plant hormones, which play multiple roles including promoting survival in the phyllosphere and manipulating host defense signaling to promote virulence (Vorholt, 2012; Chanclud and Morel, 2016; Patkar and Naqvi, 2017). Our knowledge of plant-pathogen interactions is advancing rapidly. The existence of yeast-like fungi in the phyllosphere of wild growing *Arabidopsis* is increasingly documented (Agler et al., 2016; Wang et al., 2016; Regalado et al., 2020; Brachi et al., 2021; Almario et al., 2022), but *Arabidopsis* interactions with this class of fungi are currently not well understood.

We address this utilizing *Arabidopsis* and the *Arabidopsis*-associated yeast-like fungal species, *P. arabidopsidicola* strain C29 (Wang et al., 2021). The objective of this study is to address the influence of this resident yeast on plant immunity. These results introduce a novel experimental system involving a naturally occurring phyllosphere yeast and the host from which it was isolated, the genetic model plant *Arabidopsis*.

## Materials and methods

### Microbial strains and culture

A culture of the *P. inouyei* type strain YB-4354 was provided by the ARS culture collection.^1^ *Protomyces arabidopsidicola* strain C29, which was isolated from wild *Arabidopsis thaliana* (*Arabidopsis*; Wang et al., 2016) and subsequently described and named (Wang et al., 2021), has been deposited in the following culture collections; HAMBI, HAMBI3697 and DSMZ, DSM 110145. For the current study, all *Protomyces* species were purified twice from single colonies and cultured on yeast, peptone, dextrose (YPD; 0.5, 0.5, and 2%, respectively) agar (1.5%) at 21°C, unless otherwise indicated. *Botrytis cinerea* strain BO.510 was obtained from the lab of Tapio Palva, Helsinki, Finland. *Botrytis* growth and infection was performed as previously described (Cui et al., 2016; Vuorinen et al., 2021). Briefly, *Botrytis* was cultivated on potato dextrose broth (PDB) with 15 g/l agar in the dark at room temperature. Mycelium with conidia was harvested with forceps, suspended in 0.5× PDB, mixed vigorously, filtered through miracloth, and diluted for infections. *Botrytis* spore density was determined microscopically using a hemocytometer. A *Paenibacillus* strain, obtained from Ansa Palojärvi at the University of Helsinki, is known to produce antifungal compounds active against *Botrytis* and was used as a positive control for direct microbe-microbe interactions. Co-cultivation of *Botrytis* with *P. arabidopsidicola* and the *Paenibacillus* strain was performed on YPD agar plates at 21°C.

### *Arabidopsis* growth, infection, and trypan blue staining

Wild type Columbia-0 (Col-0) accession seeds of *Arabidopsis* were from the Nottingham Arabidopsis Stock Centre (NASC).^2^ The local accession Kivikko (Kvk1) was collected at the site where *Arabidopsis*–associated yeast isolation samples were previously collected (Wang et al., 2016). Standard conditions for *Arabidopsis* cultivation were as follows. Seeds were sown at high density on wet vermiculite peat mix (1,1, with type B2 peat; Kekkilä; www.kekkila.fi). Seeds were stratified in the dark at 4°C for 72 h, before transfer to a growth chambers (Fitotron SGC120, Weiss Technik)^3^ with 12/12 h day/night photoperiod with 170 μmol m^−2^ s^−1^ illumination, at 23°C/18°C and 65%/75% relative humidity. One-week-old seedlings were transplanted one per pot in 6 cm square pots or five per pot in 10 cm square pots with the same soil mixture. Three-week-old plants were used for infection experiments. Standard infection conditions were done using drop infections by pipetting 5 μl of an OD_600_ = 1 cell suspension of *P. arabidopsidicola* in 10 mM MgCl_2_ onto the adaxial side of the leaves, left from the midvein. Infected plants were evaluated for symptoms every 1–2 days for 2 weeks and photographed at 10 dpi for documentation, as needed. *P. arabidopsidicola* was monitored on and in infected *Arabidopsis* leaves by trypan blue staining. Trypan blue stain stock solution (0.05%) in lactophenol (1.1:1, glycerol:85% lactic acid: phenol) was diluted 1:2 in 95% ethanol for staining. Infected leaves were covered in trypan blue staining solution and incubated in a boiling water bath until staining solution started boiling (approx. 3 min). Samples were de-stained for 48 h in 2.5 g/ml chloral hydrate solution in water and placed in 60% glycerol for storage and mounting on slides. Samples were examined under a compound microscope (Leica Model MZ 2500)^4^ with a 10× eye piece and 20× or 100× objective and photographed with an attached photo documentation system (Leica model DFC490).

### *Protomyces* growth assays

Three-day-old cultures in YPD liquid medium were harvested and washed twice with Milli-Q (MQ) water. *Protomyces inouyei* (OD_600_ = 0.14, 5 μl/leaf) and *P. arabidopsidicola* strain C29 (OD_600_ = 0.10, 5 μl/leaf) suspensions were drop inoculated on the adaxial side of 24-day-old *Arabidopsis* leaves, aseptically grown on 0.5× Murashige and Skoog (MS) agar plates. Sterile foil on an agar plate was used as a control for non-specific surface growth. Growth in the phyllosphere was assayed with re-isolation of *Protomyces* from inoculated leaves by placing leaves in 2 ml Eppendorf tubes with 1 ml 0.025% Silwet-L77 in water and shaking (800 rpm; VWR microplate shaker) for 1 h, after which 5 μl wash solution was serial diluted and plated on YPD agar for colony counting. Pooled data from five biological repeats were analyzed using a linear mixed model with nlme package in *R* (v3.5.1).

### *Protomyces arabidopsidicola* cell wall preparation and root growth assay

For *P. arabidopsidicola* cell wall isolation, cells were cultivated in potato dextrose broth (PDB; Carl Roth) in 200 ml flasks on a rotary shaker for 4 days at room temperature. Cells were collected and washed twice in sterile MQ water, re-suspended in a small volume of sterile MQ water, and freeze-dried for 2 days. Dry cells were ground in a tissue homogenizer (Precellys 24)^5^ using 425–600 μm glass beads (5×15 s at 6,800 rpm, cooling at-20°C between runs). Cell disruption was confirmed using a compound microscope (Leica Model MZ 2500)^6^ with 40× objective. The insoluble cell walls were separated from the soluble fraction by centrifugation in MQ water at 1,000 *g* for 20 min in + 4°C. This crude cell wall preparation was then freeze dried as described above, and weighed.

Sterile *Arabidopsis* Col-0 accession seeds were stratified at + 4°C for 72 h and germinated vertically on 0.5× MS 0.8% agar plates for 4 days in a growth chamber (Fitotron SGC120, Weiss Technik)^7^ at ~170 μmol m^−2^ s^−1^ illumination, at +23/+18°C. Seedlings were transplanted to either 0.5× MS agar control plates or identical plates supplemented with 0.9 g/l *P. arabidopsidicola* cell wall preparation. Seedlings were grown in the same conditions for 10 more days, photographed, and root length measured using imageJ software.^8^

### *Protomyces arabidopsidicola* pre-treatment, *Botrytis* infection, and qPCR

Live and autoclaved *P. arabidopsidicola* cultures were harvested, washed, and suspended at OD_600_ = 1 (7 × 10^6^ CFU/ml) in sterile water. Three-week-old soil-grown *Arabidopsis* were pre-treated by spraying with one of the *P. arabidopsidicola* cell suspensions (0.2 ml/plant), or water as a control, then infected with a *Botrytis* a 3 μl drop of a 2 × 10^6^ spore ml^−1^ spore suspension 3 days after treatments with *P. arabidopsidicola* cells. Plants were grown in a chamber with 12/12 h (light/dark), 23/18°C, and 65/75% relative humidity. Pre-treated and infected plants were collected at 30 and 60 min for western blot, and at 24 h and 72 h for qPCR. Four or five plants were pooled, frozen in liquid nitrogen, and stored in –80°C. Leaf lesions were photographed at 72 h and diameters measured in ImageJ.^9^ Lesion size data was statistically analyzed with scripts in *R* (v3.5.1) using the nlme package. The model contrasts were estimated with multcomp package, and the estimated *p*-values were subjected to single-step correction. RNA isolation and quantitative reverse transcriptase PCR (qPCR) were performed as previously described (Cui et al., 2016). Three genes *TIP41*, *YLS8* and *PP2AA3*, which have stable expression levels in a wide variety of conditions (Czechowski et al., 2005), were simultaneously used as reference genes and are listed in (Supplementary Table S1). Stability of multiple reference gene expression and raw cycle threshold values were analyzed with Qbase+2.1 (Hellemans et al., 2007). Expression levels relative to the multiple reference genes using three technical replicates with correction for primer efficiencies and error propagation were first calculated in Qbase+2.1 (Hellemans et al., 2007) and then relative expression was calculated to facilitate comparisons within but not between the two experiments; i.e. in the yeast treatment experiment, treatment values were normalized to the 24 h control and in the yeast pre-treatment with *Botrytis* infection experiment, treatment values were normalized to the *Botrytis*-treated 24 h control. Primers used and their primer efficiencies are listed in the supplementary materials (Supplementary Table S1). Significance of differential expression was estimated by glht package in *R* (3.5.1) using false discovery rate adjustment of p-values.

### Protein extraction, SDS-PAGE, and western blotting

Total protein was isolated from 100 mg frozen rosettes in 100 μl Lacus buffer (Brader et al., 2007). Protein concentration determined by Bradford assay^10^ with BSA as standard. Total protein (100 μg) was loaded in SDS-PAGE, transferred to a membrane (PVDF type; Immobilon–FL), and scanned with LI-COR Odyssey scanner. Equal loading was monitored by amido black staining. Primary anti-TEpY (Phospho-p44/42 MAPK (Erk1/2) Thr202/Tyr204; CST^®^) rabbit monoclonal antibody specific for the activated form of MAPKs was used at a 1:2,000 dilution and the secondary anti-rabbit IgG antibody (IRDye, 800CW Goat anti-Rabbit) at 1:10,000.

### Experimental field infections

Plants for long-term field infections were sown and germinated in September in a covered cage under ambient conditions using seeds of the accessions Col-0 and a local accession Kvk1. Seven-day-old seedlings were transplanted 5 per pot in 10 cm square plastic pots with a 1:1 mix of peat and vermiculite and further grown under the covered cage under ambient conditions. Pots containing two-week-old plants were buried in sand in the Helsinki University Viikki campus experimental field in a triplicate block design at three different sites in the field and spray inoculated with water (mock) or *P. arabidopsidicola* cell suspensions (OD_600_ = 0.1 and 1.0, *ca.* 0.5 ml per pot). The field site was not pre-tested for the presence of the yeast, which is assumed to be wind dispersed and possibly present. Plants remained in the field over the course of the next 7 months (until early May), with snow cover for about 4 months, and were visually examined and photographed at various times. In May, final photos and visual examinations were done and representative mock control and infected plants were sampled for yeast isolations, performed as previously described (Wang et al., 2016). Briefly, the identity of reisolated yeasts were confirmed as *P. arabidopsidicola* by cleaved restriction fragment length polymorphism analysis of ITS PCR fragments using Taq I.

## Results

### *Protomyces arabidopsidicola* strain C29 is not pathogenic on *Arabidopsis*

**Figure 1:**
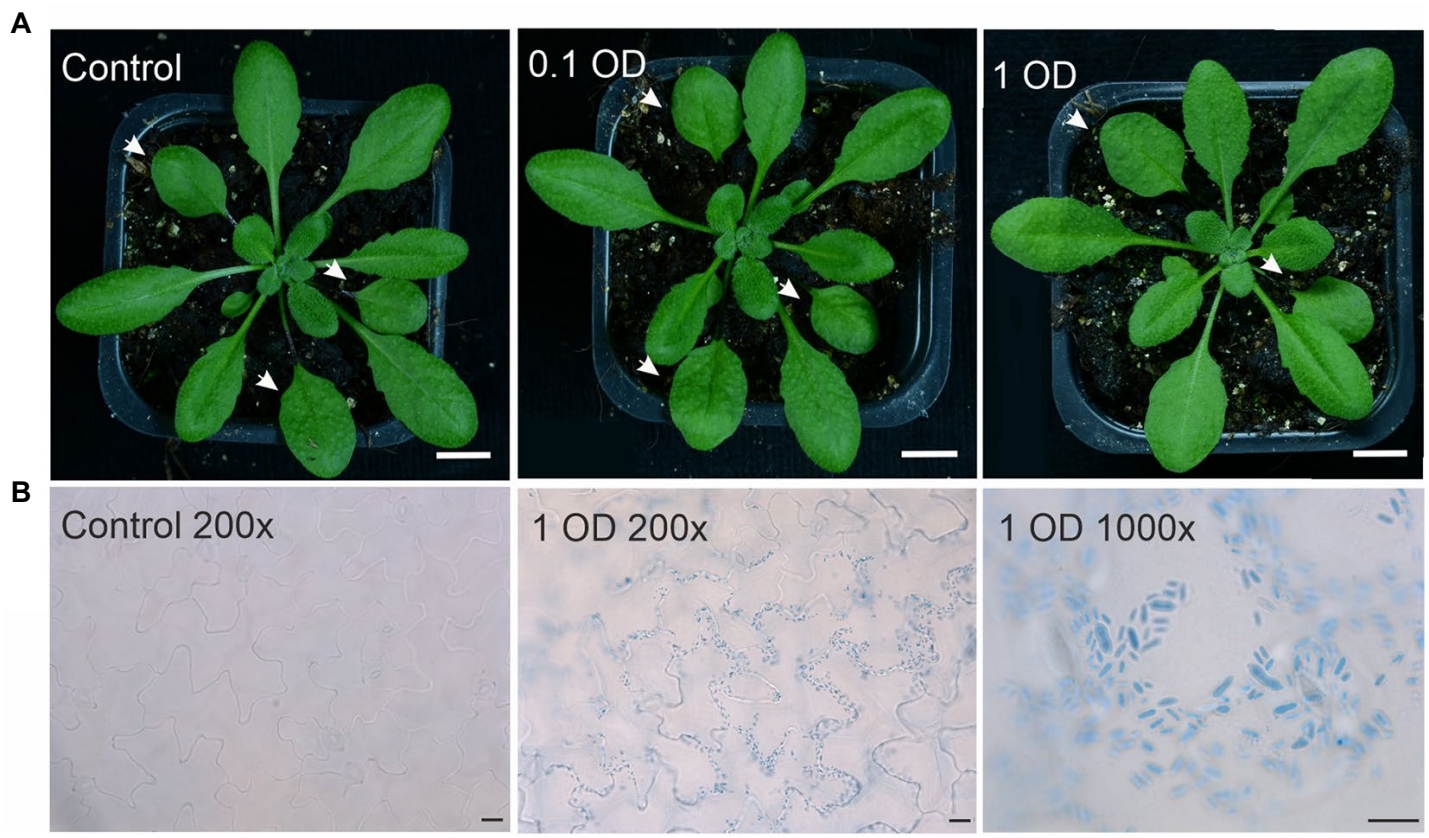
*Arabidopsis* leaves infected with *Protomyces arabidopsidicola*. **(A)** *Arabidopsis* (Col-0 accession) plants either mock treated with 10 mM MgCl_2_ (control) or drop infected in the middle of the left hand leaf half with a 0.1 OD or a 1 OD suspension of *P. arabidopsidicola* cells in 10 mM MgCl_2_ and photographed at ten days post infection. Treated leaves are indicated with white arrows. White scale bars in all photos are 1.0 cm. **(B)** Photomicrograph of *Arabidopsis* leaves either mock treated with 10 mM MgCl_2_ (control 200×) in the right panel or infected with a 1 OD suspension of *P. arabidopsidicola* cells (1 OD 200×) in the middle panel. Leaves were stained with trypan blue to visualize *P. arabidopsidicola* cells. The right hand panel (1 OD 1,000×) is a close up showing the typical *Protomyces* yeast-type cell structure of the visualized cells. Scale bars: left 20 μm, middle 20 μm, right 10 μm. All experiments used 21-day-old soil-grown *Arabidopsis* plants. Experiments were repeated 2–3 times with similar results and representative results are shown.

The genome of *P. arabidopsidicola* strain C29 harbors a heterothallic MAT locus of the P-type suggesting that it should not be able to infect *Arabidopsis* as a single strain (Wang et al., 2019). To confirm that strain C29 was not able to cause disease on *Arabidopsis*, infected plants were examined using many *Arabidopsis*-infection protocols, including chamber experiments at low temperatures and long-term field infections (Table 1), which mimicked the environmental conditions, from which *P. arabidopsidicola* was isolated. The local *Arabidopsis* accession, Kvk1, which was collected at the site where *P. arabidopsidicola* was isolated, and the accession Col-0 were used for infections. In field experiments, *P. arabidopsidicola* was recovered from infected plants, but not control plants that were mock infected with water. In all field and chamber experiments, infected *Arabidopsis* leaves were examined for formation of typical disease symptoms caused by *Protomyces* species, such as galls and tumors, as well as visible necrosis, chlorosis, and other more general disease symptoms. As expected, *P. arabidopsidicola* infection with the single strain C29, did not result in any disease symptoms on Kvk1 (not shown) or Col-0 (Figure 1A), which was representative of results obtained with both accessions. These results (Figure 1) were obtained with plants grown in chambers under standard growth conditions, but are typical for all experiments, as disease symptoms were not found under any of the tested conditions (Table 1). In field experiments, some damaged leaves were present. However, this damage was also found in control plants, suggesting they are not specific to infected plants and due to growth conditions. The location and morphology of *P. arabidopsidicola* cells was examined in infected *Arabidopsis* leaves by trypan blue staining (Figure 1B). *P. arabidopsidicola* was not observed in control uninfected plants, but clearly visible on the leaf surface of inoculated plants in its non-infectious yeast form, especially concentrated in the depressions between epidermal pavement cells (Figure 1B, center panel). Examination under high magnification confirmed the typical *Protomyces* yeast cell type morphology and no infectious hyphal cell types were found (Figure 1B, right panel). These findings support that *P. arabidopsidicola* strain C29 was a non-pathogenic resident yeast in the *Arabidopsis* phyllosphere. In field infections, *P. arabidopsidicola* survived overwinter in the *Arabidopsis* phyllosphere and was re-isolated the following spring only from plants that were inoculated in autumn with *P. arabidopsidicola*.

**Table 1:** Methods used to test infection *of Arabidopsis* with *P. arabidopsidicola* strain C29^a^.

Environment	Method^b^	Concentration^c^	Age (weeks)^d^	Temperature	Duration^e^
Growth chamber	Drop	OD = 1	2	8°C	21 d
Growth chamber	Drop	OD = 1	2	23/18°C	21 d
Growth chamber	Drop	OD = 1	4	8°C	7 d
Growth chamber	Drop	OD = 1	4	23/18°C	7 d
Growth chamber	Spray	OD = 0.1	2	8°C	21 d
Growth chamber	Spray	OD = 0.1	2	23/18°C	21 d
Growth chamber	Spray	OD = 0.1	4	8°C	7 d
Growth chamber	Spray	OD = 0.1	4	23/18°C	7 d
Growth chamber	Spray	OD = 1	2	8°C	21 d
Growth chamber	Spray	OD = 1	2	23/18°C	21 d
Growth chamber	Spray	OD = 1	4	8°C	7 d
Growth chamber	Spray	OD = 1	4	23/18°C	7 d
Growth chamber	Infiltration	OD = 0.1	4	8°C	7 d
Growth chamber	Infiltration	OD = 0.1	4	23/18°C	7 d
Field	Spray	OD = 0.1	2	Snow cover	~7 mo
Field	Spray	OD = 1	2	Snow cover	~7 mo

a The outdoor field tests utilized the *Arabidopsis* Col-0 accession and a local accession Kivikko (Kvk1) from the site where yeast isolation samples were collected. *Protomyces arabidopsidicola* strain C29 cells were used as inoculum in all experiments.

b Yeast cell suspensions were applied by pipette in 5 μl drops (Drop), or with a sprayer on the adaxial side of *Arabidopsis* leaves (Spray), or hand infiltrated into the abaxial side of *Arabidopsis* leaves using a needleless syringe (Infiltration).

c A cell density of 1 OD is equal to 7 × 10^6^ CFU/ml.

d Plant age at time of infection.

e Duration: length of time the infection was followed. wks, weeks, d, days, mo, months.

### *Protomyces arabidopsidicola* persists in the *Arabidopsis* phyllosphere

**Figure 2:**
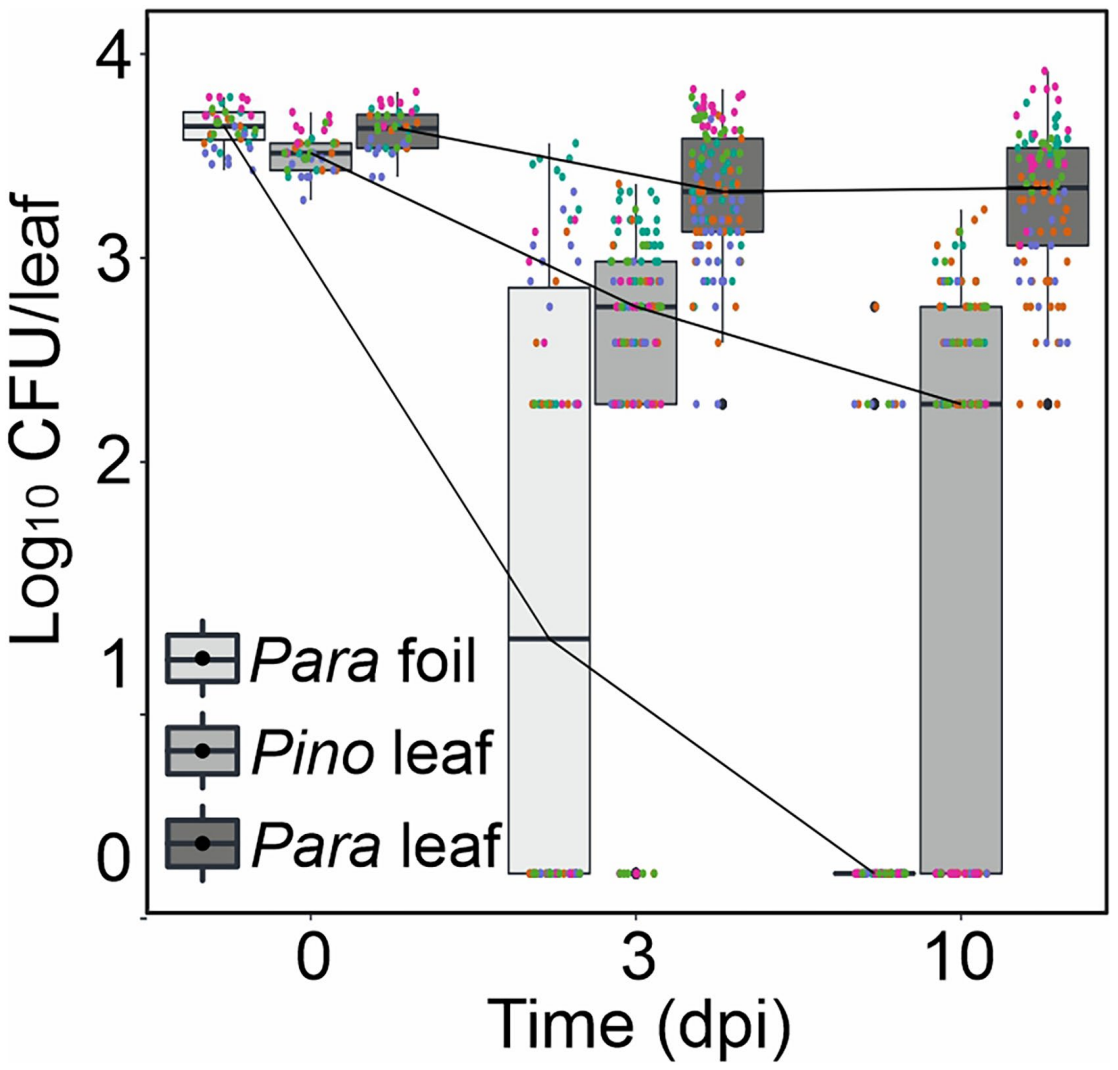
*Protomyces arabidopsidicola* strain C29 persistence on *Arabidopsis*. Persistence assay of *P. arabidopsidicola* (Para) and *P. inouyei* (Pino) on the surface of *Arabidopsis* leaves aseptically grown on 0.5 x MS agar. Cells were re-isolated from drop inoculated plants at the indicated times and plated to determine cell numbers, which are presented as the number of colony forming units (Log^10^ CFU/leaf). Persistence on sterile foil was used as a control for the ability to survive non-specifically on surfaces. Pooled data from five independent biological repeats (n = 25) were analyzed by computing a linear mixed model in *R* (v3.5.1). The pooled data is displayed in standard boxplots, all data points are plotted with a different color for each independent biological repeat.

Since *P. arabidopsidicola* was able to overwinter in the *Arabidopsis* phyllosphere under field conditions, *P. arabidopsidicola* and its most closely related species, *P. inouyei,* were tested for the ability to persist on the leaf surface of aseptic *in vitro* grown *Arabidopsis*. In these experiments, a high level of drop-inoculated *P. arabidopsidicola* persisted on the *Arabidopsis* leaf surface, with a stable level of CFUs maintained over a 10-day period (Figure 2). All recovered microbes cultivated from infected plants had typical colony characteristics of *P. arabidopsidicola* and control plates (not shown) were free of microbes. The *Arabidopsis* phyllosphere supported a similar but slightly lower level of *P. inouyei,* which is a close relative *P. arabidopsidicola*. At the 10 days post infection, the level of *P. arabidopsidicola* was only about 10-fold higher than that of *P. inouyei*. This demonstrated that over the time-period studied, the ability to persist in the *Arabidopsis* phyllosphere was common to both *P. arabidopsidicola* and *P. inouyei*. Further, as a negative control, *P. arabidopsidicola* survival was tested on sterile foil placed inside the same agar medium plates. After 10 days, the level of recovered CFUs dropped to near zero, indicating it was not able to persist on surfaces non-specifically (Figure 2).

### *Protomyces arabidopsidicola* immunity priming on *Arabidopsis*

**Figure 3:**
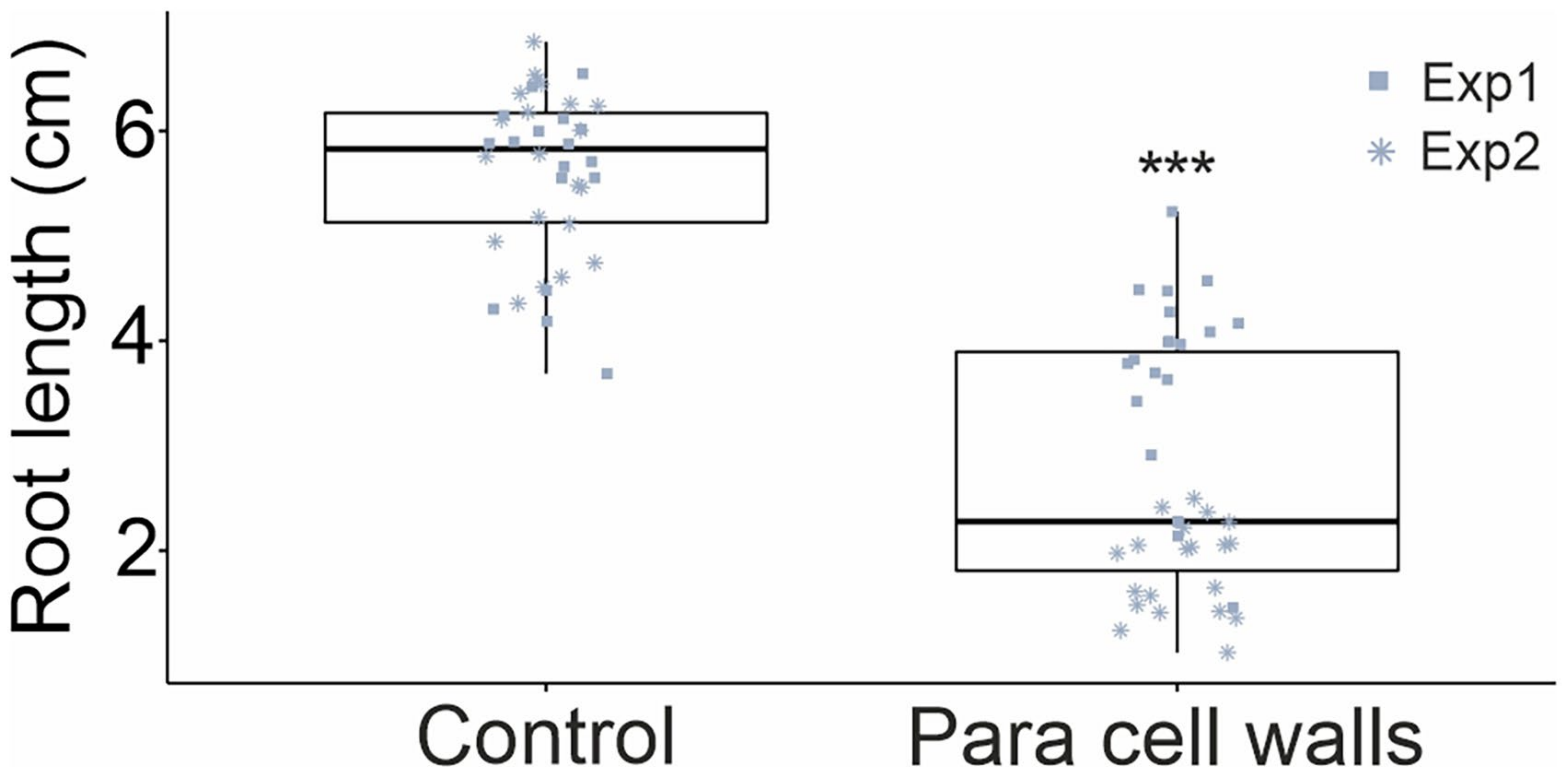
Inhibition of *Arabidopsis* growth by *P. arabidopsidicola* cell wall preparations. *Arabidopsis* growth was quantified in an *in vitro* root growth assay that measured root length of 14-day-old seedlings that had been grown for 10 days on vertically oriented 0.5× MS agar plates without (Control) or with addition of 0.9 g/l *P. arabidopsidicola* cell walls (Para cell walls). The experiment was replicated in two independent biological repeats and pooled data is presented (*n* = 19 in each repeat, total *n* = 38). Statistical significance was tested using two-way ANOVA in *R* (v3.5.1), the treatment effect was significant (^***^p = 2 × 10^−16^). The pooled data is displayed in standard boxplots; all data points are plotted with a different shape for each independent biological repeat.

The activation of defenses and growth are finely balanced and mutually inhibitory; accordingly, activation of immune defense responses results in a well-known effect where growth is inhibited (Belkhadir et al., 2014; Huot et al., 2014). This effect was used to explore the activation of *Arabidopsis* immune signaling by *P. arabidopsidicola*. In *in vitro* root growth assays, including *P. arabidopsidicola* cell wall preparations in the growth medium resulted in significant inhibition of root growth (Figure 3). This result supports that *P. arabidopsidicola* cell walls contain immune stimulating molecular patterns.

**Figure 4:**
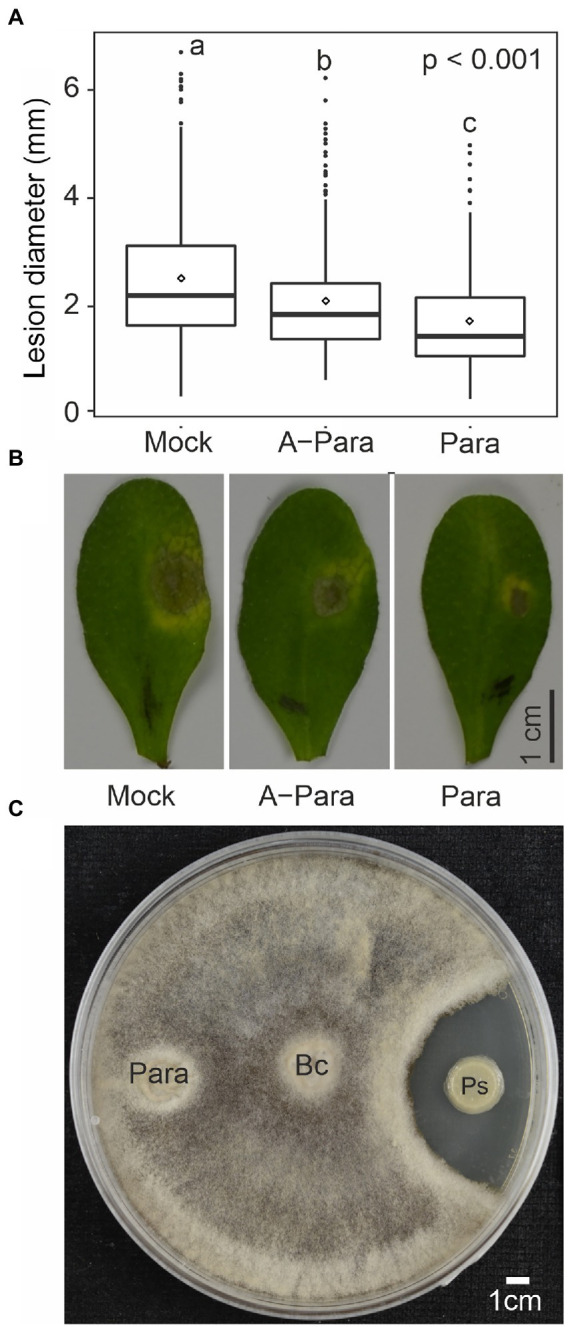
Priming effect by *P. arabidopsidicola*. **(A)** Lesion diameters from drop infection of *Botrytis* spores on leaves of *Arabidopsis* pre-treated with water (mock), autoclave killed *Protomyces arabidopsidicola* C29 (A-Para), or live Para. The pooled data from six independent biological repeats (total *n* = 24) is displayed in standard boxplots (central 50% of the data range in the box, with a line showing the median, bars depict the range of remaining data, and dots display outlier data points). Statistics performed with pooled data from by computing a linear mixed model in *R* (v3.5.1). **(B)** Photos of *Botrytis*-induced necrosis on leaves of *Arabidopsis* that were pre-treated with water (mock), autoclave killed Para (A-Para), or live Para. **(C)** No direct growth inhibition by *P*. *arabidopsidicola* strain C29 (Para) was observed against *Botrytis cinerea* (Bc) in co-cultivation assays. As a control, a *Paenibacillus* sp. (Ps) known to have antifungal activity against *Botrytis* was used. Yeast extract-peptone-dextrose agar plates were used for cultivation. Photos were taken 7 days post inoculation. Experiment was repeated three time with similar results and one representative result is shown.

To further explore if *P. arabidopsidicola* can engage *Arabidopsis* immunity, the ability of strain C29 to alter resistance against a broad-host-range fungal pathogen was tested. Plants were pre-inoculated with water, a suspension of live strain C29 cells, or a suspension of strain C29 cells killed by autoclaving. Plants were then infected with the necrotrophic fungal pathogen *Botrytis* and disease progression monitored as the size of *Botrytis*-induced spreading lesions. Significantly smaller lesion diameter was observed in *Arabidopsis* pre-treated with both live and killed *P. arabidopsidicola* cell-suspensions (Figures 4A,B), suggesting increased resistance. Additionally, plants pre-treated with live *P. arabidopsidicola* cells had significantly smaller lesions than those treated with dead cells (Figures 4A,B). This suggests that yeast PAMP molecules liberated from dead *P. arabidopsidicola* cells could trigger plant immunity, but the full effect required live cells. In order to test for direct microbe-microbe interactions, *Botrytis* and *P. arabidopsidicola* were co-cultivated on PDB agar, a medium known to support growth of both of these fungi. This demonstrated that *P. arabidopsidicola* cultures were not able to inhibit *Botrytis* growth *in vitro* (Figure 4C), arguing against the possible direct antagonistic effect by *P. arabidopsidicola*. These results suggest *P. arabidopsidicola* on the *Arabidopsis* leaf surface may inhibit *Botrytis* disease spread *via* interaction with the plant to activate immune signaling, rather than *via* direct microbe-microbe interaction.

### *Protomyces arabidopsidicola* activates MAPK and immune signaling pathways

**Figure 5:**
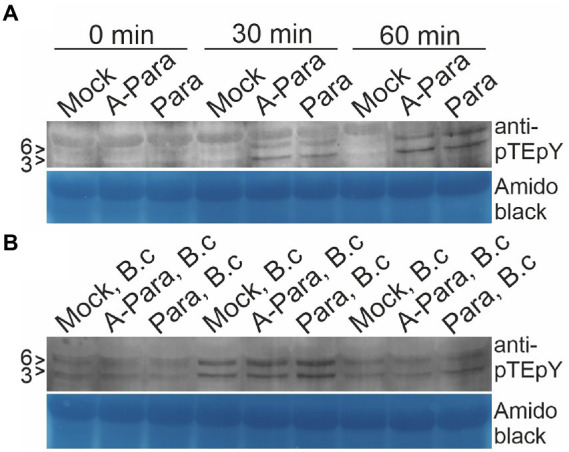
*Arabidopsis* MAPK activation by *P. arabidopsidicola*. *Arabidopsis* mitogen activated protein kinase (MAPK)3 and MAPK6 (MAPK3/6) activation was monitored by western blot with anti-phospho MAPK antibodies following pre-treatment with autoclaved *P. arabidopsidicola* strain C29 (A-Para) or live *P. arabidopsidicola* strain C29 (Para). The positions of the 43 and 45 kDa AtMPK3 and AtMPK6 are indicated to the left of the protein blots with the respective markers (3 > and 6 >). MAPK3/6 were activated by both A-Para and Para pre-treatment at 30 and 60 min. **(A)** Leaf samples were collected at 30 and 60 min after A-Para or Para pre-treatment. **(B)** Leaf samples were collected at 30 and 60 min after *Botrytis* infection was applied to plants which has been pre-treated with A-Para or Para for 3 days. Equal loading was confirmed by amido black staining. Three independent experiments were repeated with the same result, of which representative results are shown. B.c: *Botrytis cinerea*.

Mitogen-activated protein kinases (MAPK) activation is among the earliest events in *Arabidopsis* immune responses. MAPK activation in response to pre-treatment with *P. arabidopsidicola* cells was assayed, both alone and with subsequent *Botrytis* infection. Western blots probed with the pTEpY antibody, specific for the active phosphorylation site of MAPKs, revealed slight activation of *Arabidopsis* MAPK3 and MAPK6 (MAPK3/6) in response to treatment with both live or dead *P. arabidopsidicola* cells at 30 min and this response increased to a higher level by 60 min (Figure 5A). When plants were infected with *Botrytis*, at 30 min all samples exhibited MAPK3/6 activation, which had returned to near the control level by 60 min (Figure 5B). Some mock-infection controls showed moderate MAPK-activation, likely due to slight wounding during the mock treatment. Plants pre-treated with live *P. arabidopsidicola*, dead *P. arabidopsidicola*, or mock treatment showed a similar degree of MAPK3/6 activation at 30 min after *Botrytis* infection. We conclude that single treatments with live or dead *P. arabidopsidicola*, or *Botrytis*, rapidly activated MAPK3/6 in *Arabidopsis.*

**Figure 6:**
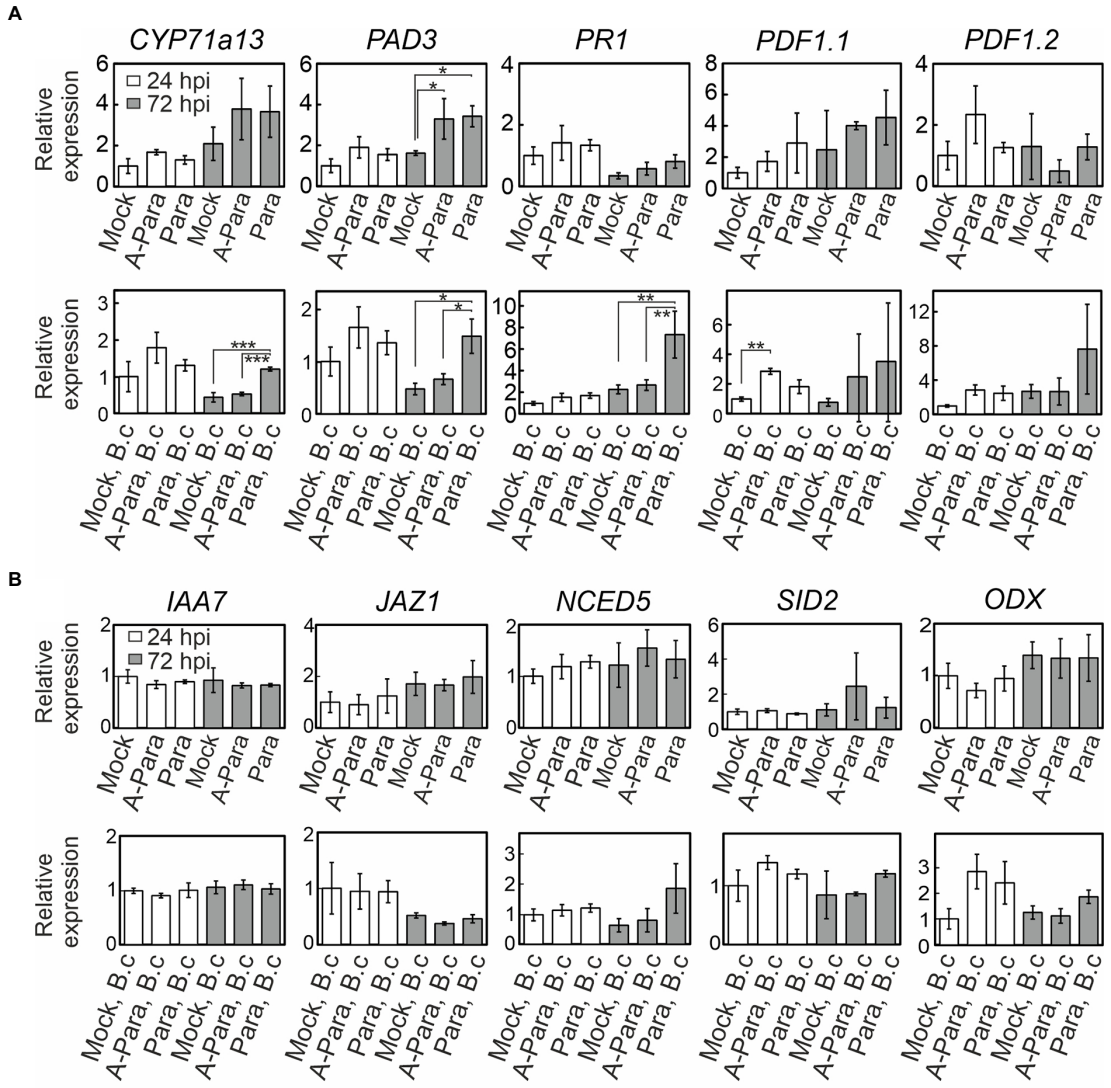
Plant defense and hormone signaling marker expression. Relative gene expression of plant defense and hormone signaling genes at 24 (white bar) and 72 (grey bar) hours post infection (hpi) following *P. arabidopsidicola* strain C29 treatment only (top row), and with *Botrytis* infections following strain C29 pre-treatments (bottom row). Relative expression is calculated to facilitate comparisons within but not between the top and bottom rows; i.e. top row is normalized to the 24 h control and the bottom row is normalized to the *Botrytis*-treated 24 h control. All time points are relative to the last treatment; relative to the strain C29 treatment in the top row and relative to the *Botrytis* treatment in the bottom row. **(A)** Expression of the genes: *CYP71a13*, *CYTOCHROME P450, FAMILY 71, SUBFAMILY A, POLYPEPTIDE 13,* a marker for camalexin biosynthesis*; PAD3, PHYTOALEXIN DEFICIENT 3,* a camalexin marker*; PR1, PATHOGENESIS-RELATED GENE 1,* a late salicylic acid-response marker*; PDF1.1, PLANT DEFENSIN 1.1,* a jasmonic acid-response marker*; PDF1.2*, *PLANT DEFENSIN 1.2*, a jasmonic acid-response marker. **(B)** Expression of the genes: *IAA7, INDOLE-3-ACETIC ACID 7*, an auxin-response marker; JAZ1, *JASMONATE-ZIM-DOMAIN PROTEIN1*, an early jasmonic acid-response marker; NCED5, *NINE-CIS-EPOXYCAROTENOID DIOXYGENASE 5*, an ABA-response marker; SID2, *SALICYLIC ACID INDUCTION DEFICIENT 2*, a salicylic acid-response marker; ODX, *2-OXOACID-DEPENDENT DIOXYGENASE*, a salicylic acid-response marker. Data are presented as mean relative expression levels ± SD of three pooled biological repeats. Statistics was performed with glht package in *R* (v3.5.1) with fdr method for adjusted *p* values. Para: *P. arabidopsidicola* strain C29; A-Para: autoclaved *P. arabidopsidicola* strain C29; B.c: *Botrytis cinerea* infected. ^*^p < 0.05; ^**^p < 0.01; ^***^p < 0.001.

To further explore activation of defense signaling, qPCR was employed to monitor expression of defense signaling marker genes (Supplementary Table S1). Strikingly, evidence of enhanced activation of immune signaling was observed. This was seen as enhanced *Botrytis*-induced transcriptional responses, in samples pre-treated with live or dead *P. arabidopsidicola* cells, for *PDF1.1,* a JA marker; *CYP71a13* and *PAD3*, markers of camalexin, which is the primary antimicrobial compound in *Arabidopsis*; and *PR1*, a marker of the late SA response (Figure 6A). Live or dead *P. arabidopsidicola* cells alone had limited effect on defense signaling activation, showing induction of *PAD3* at 72 hpi (Figure 6A). Taken together, the all the results presented above indicate *P. arabidopsidicola* cells had immunity priming effect in *Arabidopsis*, and activated some of the known canonical defense pathways that involve JA, SA, and camalexin. Markers for other hormone signaling pathways that have also been implicated in immune signaling, such as ethylene, ABA, and auxin, exhibited no significant changes (Figure 6B), suggesting the response to treatment with *P. arabidopsidicola* is independent of these pathways.

## Discussion

In this study, we aimed to establish an experimental system for the study of immune system interaction between the genetic model plant *Arabidopsis* and a naturally occurring phyllosphere resident yeast-like fungus. *Protomyces arabidopsidicola* has been found in virtually all studies addressing fungal residents of the *Arabidopsis* phyllosphere (Agler et al., 2016; Wang et al., 2016, 2019; Regalado et al., 2020; Brachi et al., 2021). The occurrence of *P. arabidopsidicola* on wild and field grown *Arabidopsis,* together with its ability to persist in the *Arabidopsis* phyllosphere, both under growth chamber and experimental field conditions, support that this yeast is naturally associated with *Arabidopsis* and can utilize its phyllosphere as a living space.

The question of specificity in this yeast-plant interaction remains only partially resolved. *Protomyces inouyei,* a close relative of *P. arabidopsidicola*, was also able to persist in *Arabidopsis* phyllosphere in *in vitro* experiments, but to a lesser extent. This suggests that the other closely related *Protomyces* species, *P. inouyei*, *P. lactucaedebilis*, and *P. pachydermus*, which form a distinct clade with *P. arabidopsidicola* (Wang et al., 2019, 2021), may be able to grow on *Arabidopsis*, at least on aseptic *in vitro grown* plants. Further persistence assays and growth assays with a broader selection of *Protomyces* species will be needed in future studies to test this. However, there are many surveys of *Arabidopsis* phyllosphere yeasts that have identified *P. arabidopsidicola*, but none of these studies found any of the other above mentioned *Protomyces* species (Agler et al., 2016; Wang et al., 2016, 2019; Regalado et al., 2020; Brachi et al., 2021; Lind and Pollard, 2021; Almario et al., 2022). This suggests that *P. arabidopsidicola* could be better adapted to the *Arabidopsis* leaf surface in the wild and further implies that factors other than the host, possibly the host microbiome, participate in limiting the ability of yeasts to survive in their host phyllosphere. Accordingly, study of the *Arabidopsis* phyllosphere microbiome reveals the formation of complex functionally interrelated microbial communities (Agler et al., 2016; Chaudhry et al., 2021; Almario et al., 2022). *Protomyces arabidopsidicola* was also found in a number of other environmental samples, including several plant species, such as bean and cereal crop-related wild grasses (Prior et al., 2017; Wang et al., 2019; Sun et al., 2020; Wang et al., 2021), supporting that it can also survive in the phyllosphere of plants other than *Arabidopsis*. Although *P. arabidopsidicola* may not be specific to only *Arabidopsis*, the current study still illustrates a broadly distributed yeast living in the phyllosphere of multiple plants with the ability to modulate the health of *Arabidopsis* and potentially other plants. This suggests that work with *P. arabidopsidicola* may be relevant beyond just the genetic model plant *Arabidopsis*.

The nature of this yeast–plant interaction also requires further clarification and confirmation. Nearly all *Protomyces* species are heterothallic (Reddy and Kramer, 1975; Kurtzman, 2011; Wang et al., 2019), and thus require a strain of the opposite MAT type in order to enter the phytopathogenic hyphal state. Genome sequencing has confirmed MAT loci consistent with heterothallism in six of seven *Protomyces* species examined (Wang et al., 2019). The presence of a heterothallic MAT locus of the *P*-type within the genome of *P. arabidopsidicola* strain C29 (Wang et al., 2019) predicts that it cannot infect *Arabidopsis* as a single strain, i.e., in the absence of a second strain of the compatible *M* mating type. Results from *Arabidopsis* infection experiments presented here confirm this prediction. Further isolations to obtain *matM* type strains of *P. arabidopsidicola* and then coinfection tests with two strains of compatible MAT types infecting multiple different accessions will be required to test if *P. arabidopsidicola* is pathogenic on *Arabidopsis*. Regardless of the outcome of these tests, the *P. arabidopsidicola*–*Arabidopsis* system remains relevant; either for testing interactions with a non-pathogenic phyllosphere resident, or the very interesting prospect of testing the interactions of a host plant with a latent pathogen that resides as a single strain in the phyllosphere while it waits for a compatible mating partner.

The findings presented here illustrate the utility of this new experimental system. Importantly, this work demonstrates the ability of a native phyllosphere resident yeast to activate *Arabidopsis* defense signaling and induce immunity against *Botrytis*. This provides evidence supporting the existence of ecologically relevant yeast PAMPs that trigger plant immunity. The human innate immune system deploys a distinct set of PRRs activated by mannose containing linkages that are highly abundant in the outer layers of cell walls of human pathogenic yeasts, such as *Candida albicans* (Jouault et al., 2009; Saijo et al., 2010). Plant pattern-recognition receptors that perceive yeast PAMPs remain largely unknown, although many studies demonstrate the ability of yeasts to activate plant immune signaling. For example, yeast cell wall extract of the budding yeast *Saccharomyces pastorianus* activates JA and azelaic acid signaling to induce plant disease resistance (Yaguchi et al., 2017). In *Arabidopsis*, yeast cell wall extract activates the JA, ethylene, and SA signaling pathways, and SA accumulation and signaling were required for induced resistance (Narusaka et al., 2015). Autoclaved cell suspensions of *S. cerevisiae* also engaged *Arabidopsis* immune signaling; specifically, SA signaling, camalexin biosynthesis, and enhanced resistance against other pathogens (Raacke et al., 2006). Accordingly, in the current study, *P. arabidopsidicola* cell walls caused root growth inhibition and live cell suspensions primed the SA- and camalexin-signaling pathways, enhanced *Botrytis* resistance, and activated MAPK signaling. MAPK activation induced by some PAMPs is quite rapid and transient; however activation kinetics can be quite varied depending on which MAPK is activated and the activation stimuli (Bigeard and Hirt, 2018). Our study tracked MAPK3 and MAPK6 activation at 30 and 60 min, which is well within known kinetics for these MAPKs.

*Protomyces* species are well known to produce auxin (Streletskii et al., 2016); including *P. arabidopsidicola,* where this was demonstrated with multiple lines of evidence. *Protomyces arabidopsidicola* accumulated indolic compounds in culture filtrates that activated *Arabidopsis* auxin signaling assessed by expression of the auxin responsive DR5::GUS promoter-reporter system and by root bioassays (Wang et al., 2016, 2019). Analysis of the *P. arabidopsidicola* genome also revealed a full pathway for IAA biosynthesis *via* the indole-3-pyruvic acid pathway (Wang et al., 2019). Remarkably, infection of *Arabidopsis* with *P. arabidopsidicola* did not result in activation of the auxin responsive marker gene *IAA7*. Auxin of microbial origin is multifunctional; acting in the microbe to control development, promote phyllosphere survival, in microbe–microbe interactions, and in the host plant, for instance to suppress defense responses (Spaepen et al., 2007). Thus, future studies will be required to further test for *in planta* IAA production by *P. arabidopsidicola* and understand its potential functions.

Live *P. arabidopsidicola* pre-treatment lead to significantly greater immunity against *Botrytis* than pre-treatment with dead (autoclaved) cells, supporting that live *P. arabidopsidicola* cells are required for the full priming effect. The ability of dead cells to illicit these responses supports the involvement of PAMPs, which are passive and do not require active metabolism for their activity. However, there are alternative explanations that may account for the higher priming by live cells. The persistence of living yeast cells might provide prolonged stimuli and thus stronger priming effects. Additionally, small secreted proteins/peptides from live *P. arabidopsidicola*, which contains large number of effector candidates (Wang et al., 2019), might be involved in this stronger priming. However, further studies will be required to resolve this question.

Multiple studies have previously documented enhanced *Botrytis* resistance in *Arabidopsis* pre-treated with either autoclaved or live yeast cells. Antagonistic phyllosphere microbes, including yeasts (Liu et al., 2013; Eitzen et al., 2021), can effectively suppress diseases that are caused by pathogenic fungi through a variety of known mechanisms (Legein et al., 2020; Chaudhry et al., 2021), such as direct inhibition effects, and indirect effects such as nutrient and space competition or complex microbiome interactions. In the current study, there was no obvious *Botrytis* growth inhibition by *P. arabidopsidicola* in an *in vitro* co-cultivation experiment. In comparison, the inhibitory effect of a *Paenibacillus* species known to produce antifungal compounds active against *Botrytis* was clearly observed. This suggests the enhanced immunity against *Botrytis* induced by *P. arabidopsidicola* was due to defense priming, rather than direct antagonistic effects. However, *in vitro* studies do not reflect the complex environment of the leaf, where nutrient and space competition by live *P. arabidopsidicola* and *Botrytis* might occur. Additionally, the leaf microbiome may be modified by the presence of *P. arabidopsidicola*. This activity could be mediated *via* activation of the host plant immune system or *via* indirect microbe–microbe interactions (Chaudhry et al., 2021). Notably, Brachi et al. (2021) reported an *Arabidopsis*-associated *Protomyces* strain that acts as a phyllosphere microbiome reorganizing hub species and was among the most abundant fungi in the *Arabidopsis* phyllosphere. In light of these alternate possibilities, complex indirect effects cannot be fully excluded at this time.

*Protomyces* and *Taphrina* are sister genera and share similar lifecycles and virulence strategies (Reddy and Kramer, 1975; Fonseca and Rodrigues, 2011; Kurtzman, 2011; Wang et al., 2021; Christita et al., 2022a,b). Both of these genera invade hosts in their hyphal form, which contains chitin (Valadon et al., 1962; Fonseca and Rodrigues, 2011; Kurtzman, 2011). Indeed, all fungi have chitin in their cell walls, and it is especially abundant and a major PAMP in filamentous (hyphal) fungal pathogens (Latge, 2007; Doehlemann and Hemetsberger, 2013). However, *Schizosaccharomyces pombe*, *Pneumocystis* species, and some *Taphrina* species, which all reside in the Taphrinomycotina together with *Protomyces* species, have reduced chitin content in many structures (Matsuo et al., 2004; Christita et al., 2021). Accordingly, reduced complement of chitin biosynthesis genes was also seen in a *Taphrina* strain that is pathogenic on *Arabidopsis* (Christita et al., 2021). Genomes of pathogenic fungi contain LysM domain effectors, which block chitin-induced immune responses (Kombrink and Thomma, 2013). Remarkably, many human pathogenic yeasts, whose chitin cell wall layers are buried deep beneath layers of beta-glucan and mannoproteins (Perez-Garcia et al., 2011) lack LysM effectors (Kombrink and Thomma, 2013). The sequencing of the genomes of *P. arabidopsidicola* and six other *Protomyces* species revealed an absence of the expected candidate effector proteins bearing the LysM domain (Wang et al., 2019). This suggests that they either have reduced chitin, or have their chitin layers sequestered by other structures. Effector candidates bearing the legume (L)-type lectin domain were present in all but one *Protomyces* genome (Wang et al., 2019). The L-lectin domain is known to mediate the binding of mannose linkages (Itin et al., 1996; Satoh et al., 2006, 2007). The cell wall composition has been addressed in only one *Protomyces* species. *P. inundatus* has a cell wall composed of glucan and mannose, similar to other yeasts (Valadon et al., 1962). Taken together, these results support the need for further examination of mannose-linkages, or other yeast MAMPs, in the interactions of *Protomyces* with their plant hosts. Screens aimed at discovering yeast-perceiving PRRs are underway.

## Conclusion

We present evidence that *Protomyces arabidopsidicola* strain C29 was not pathogenic on *Arabidopsis*, the premiere genetic model plant and the host from which it was originally isolated. However, it was able to persist in the *Arabidopsis* phyllosphere and activate immune signaling pathways to induce immunity against subsequent challenge with the generalist necrotrophic fungal pathogen, *Botrytis*. These finding contribute toward establishing the *P. arabidopsidicola*–*Arabidopsis* interaction as a model system, which can facilitate future genetic studies into the biology of *Protomyces* species, the evolution of fungal virulence, host interactions with phyllosphere yeasts, and plant immunity against yeasts.

## Data availability statement

The original contributions presented in the study are included in the article/Supplementary material, further inquiries can be directed to the corresponding authors.

## Author contributions

KW and AA performed the experiments. KO provided supervision and project administration. KW and KO wrote the manuscript. KW, AA, and KO were involved in designing experiments, analyzing data, and interpreting data. All authors contributed to the article and approved the submitted version.

## Funding

This work was supported by the following grants: Academy of Finland Fellowship (decisions no. 251397, 256073, and 283254) to KO and the Academy of Finland Center of Excellence in Primary Producers 2014-2019 (decisions #271832 and 307335). Lecturer Betty Väänänen grant from the Kuopio Naturalists’ Society (KLYY) fund and a grant from the August Johannes and Aino Tiura Agricultural Research Foundation to AA. KW was a member of the University of Helsinki Doctoral Program in Plant Science (DPPS) and AA has a PhD fellowship from the Microbiology and Biotechnology Doctoral Program.

## Conflict of interest

The authors declare that the research was conducted in the absence of any commercial or financial relationships that could be construed as a potential conflict of interest.

## Publisher’s note

All claims expressed in this article are solely those of the authors and do not necessarily represent those of their affiliated organizations, or those of the publisher, the editors and the reviewers. Any product that may be evaluated in this article, or claim that may be made by its manufacturer, is not guaranteed or endorsed by the publisher.

## Footnotes

^1^ https://nrrl.ncaur.usda.gov/

^2^ http://arabidopsis.info/

^3^ www.weiss-technik.com

^4^ https://www.leica-microsystems.com

^5^ https://www.bertin-instruments.com

^6^ https://www.leica-microsystems.com

^7^ www.weiss-technik.com

^8^ imagej.nih.gov/ij/

^9^ https://imagej.nih.gov/ij/

^10^ www.bio-rad.com/
