# Supplementary material for: The immunity priming effect of the Arabidopsis phyllosphere resident yeast Protomyces arabidopsidicola strain C29
Source: Front Microbiol. 2022 Sep 2;13:956018. doi: 10.3389/fmicb.2022.956018 (PMC9478198; doi:10.3389/fmicb.2022.956018)
Supplement: Supplementary file 1 [file Data_Sheet_1.PDF]

Table S1. Marker genes and primers used for quantitative real time qPCR in this study.

Gene	Full gene name	Marker for	AGI code	Primer efficiency	Forward primer	Reverse primer
CYP71a13	*CYTOCHROME P450, FAMILY 71, SUBFAMILY A, POLYPEPTIDE 13*	Camalexin	At2g30770	1.957	GGGTAGAGGCTGGACCAAAT	ACAACCGAAGATGGAAATGC
IAA7	*INDOLE-3-ACETIC ACID 7*	Auxin	At3g23050	1.85	TGACCAAAAGAGAGGAAACTTCA	AACCGACAGACACATGTTGG
JAZ1	*JASMONATE-ZIM-DOMAIN PROTEIN 1*	Early JA	At1g19180	1.801	CGTGGCTCGGTTTAGCAG	TGAAGCAACGTCGTCAAAAG
NCED5	*NINE-CIS-EPOXYCAROTENOID DIOXYGENASE 5*	ABA	At1g30100	1.77	AATTGCCGTCAAGAGTACCG	AACATATCCGCCGAATTCAC
ODX / DIN11	*2-OXOACID-DEPENDENT DIOXYGENASE / DARK INDUCIBLE 11*	SA	At3g49620	1.816	GTGGACGGTGATTGGATACC	TGGATTGGTAAACTCCGTTTG
PAD3	*PHYTOALEXIN DEFICIENT 3*	Camalexin	At3g26830	1.69	GATGTTCCTGCGAAAACACA	GTTTTGGATCACGACCCATC
PDF1.1	*PLANT DEFENSIN 1.1*	JA	At1g75830	1.924	GCTCTTGAAGCACCGATGGT	TGATTCTTGCACGCGTTACTG
PDF1.2	*PLANT DEFENSIN 1.2*	JA	At5g44420	1.698	CCAAACATGGATCATGCAAC	CACACGATTTAGCACCAAAGA
PR-1	*PATHOGENESIS-RELATED GENE 1*	Late SA	At2g14610	1.72	TGATCATGCATACACACGTACA	CATCCTGCATATGATGCTCCT
RAP2.6	*RELATED TO AP2 6*	JA	At1g43160	1.821	ACGTGTATGGCTTGGGACAT	CGGGGAAATTAAGCTTTGCT
SID2	*SALICYLIC ACID INDUCTION DEFICIENT 2*	SA	At1g74710	1.928	GCGAGGAGAGTGAATTTGCAGTCG	CCACTCTGAAGATGGGTCACTTCCA
TIP41	*AP42 INTERACTING PROTEIN OF 41 KDA*	Reference	At4g34270	1.867	GTGAAAACTGTTGGAGAGAAGCAA	TCAACTGGATACCCTTTCGCA
YLS8	*YELLOW-LEAF-SPECIFIC GENE 8*	Reference	At5g08290	1.822	TTACTGTTTCGGTTGTTCTCCATTT	CACTGAATCATGTTCGAAGCAAGT
PP2AA3	*PROTEIN PHOSPHATASE 2A SUBUNIT A3*	Reference	At1g13320	1.854	GCGGTTGTGGAGAACATGATACG	GAACCAAACACAATTCGTTGCTG
